# Childhood IgA Vasculitis (Henoch Schonlein Purpura)—Advances and Knowledge Gaps

**DOI:** 10.3389/fped.2019.00257

**Published:** 2019-06-27

**Authors:** Louise Oni, Sunil Sampath

**Affiliations:** ^1^Department of Paediatric Nephrology, Alder Hey Children's NHS Foundation Trust Hospital, Liverpool, United Kingdom; ^2^Department of Women's and Children's Health, Institute of Translational Medicine, University of Liverpool, Liverpool, United Kingdom; ^3^Department of Paediatric Rheumatology, Alder Hey Children's NHS Foundation Trust Hospital, Liverpool, United Kingdom

**Keywords:** vasculitis, immunoglobulin A vasculitis, Henoch Schonlein Purpura, child, pediatric

## Abstract

Immunoglobulin A vasculitis (IgAV; formerly Henoch Schonlein Purpura) is the most common form of childhood vasculitis. It can occur in any age and peaks around 4–6 years old. It demonstrates seasonal variation implicating a role for environmental triggers and geographical variation. The diagnosis is made clinically and 95% of patients will present with a rash, together with any from a triad of other systems—gastrointestinal, musculoskeletal, and renal. Most cases of IgAV in children have an excellent outcome. Treatment may be required during the acute phase for gastrointestinal involvement and renal involvement, termed IgAV nephritis (previously HSP nephritis), is the most serious long-term manifestation accounting for ~1–2% of all childhood end stage kidney disease (ESKD). It therefore requires a period of renal monitoring conducted for 6–12 months. Patients presenting with nephrotic and/or nephritic syndrome or whom develop significant persistent proteinuria should undergo a renal biopsy to evaluate the extent of renal inflammation and there are now international consensus guidelines that outline the indications for when to do this. At present there is no evidence to support the use of medications at the outset in all patients to prevent subsequent renal inflammation. Consensus management guidelines suggest using oral corticosteroids for milder disease, oral, or intravenous corticosteroids plus azathioprine or mycophenolate mofetil or intravenous cyclophosphamide for moderate disease and intravenous corticosteroids with cyclophosphamide for severe disease. Angiotensin system inhibitors act as adjunctive treatment for persisting proteinuria and frequently relapsing disease may necessitate the use of immunosuppressant agents. Renal outcomes in this disease have remained static over time and progress may be hindered due to many reasons, including the lack of reliable disease biomarkers and an absence of core outcome measures allowing for accurate comparison between studies. This review article summarizes the current evidence supporting the management of this condition highlighting recent findings and areas of unmet need. In order to improve the long term outcomes in this condition international research collaboration is urgently required.

## Introduction and Epidemiology

Immunoglobulin (Ig) A vasculitis [IgAV; formerly known as Henoch Schonlein Purpura (1)] is the most common form of childhood vasculitis. It is a non-thrombocytopenic, small vessel vasculitis that typically presents acutely. IgAV is by far the most frequently encountered childhood vasculitis with an incidence of 3–27 cases per 100,000 child population (2, 3). It is therefore seen regularly by pediatricians. It can present in any age, even during adulthood, but it is much more frequently seen in childhood and as such the age at peak incidence is around 4–6 years old. In childhood-onset disease, 90% of cases occur under the age of 10 years. It is extremely rare in infants. In children, it has a slight male predominance (1.5:1 male: female ratio) and a decreasing incidence according to increasing age (4). Older children, mainly teenagers with IgAV, are more likely to have disease that reflects that of adult-onset IgAV (5). Adult onset disease differs from childhood onset disease in terms of its manifestations with a large comparative series describing adults rarely presenting with abdominal pain (10 vs. 37%) and adults have higher frequency of joint involvement when compared to children (90 vs. 44%) (6). Longer-term outcomes are generally good and appear similar between the two age groups (7) although smaller reports suggest that adults are more likely to progress to end stage kidney disease (ESKD) (8). IgAV can occur in any race and it predominates in certain parts of the world such as Korea and Japan.

## Pathophysiology

Very little is understood about the exact pathophysiology of this condition except that it is felt to be directed by abnormal immunoglobulin A (IgA) and hence the recent change in nomenclature (1). IgA is a major class of antibody that is present in the mucosal secretions and it is the key first line of defense against invasion by pathogens. In the kidney, IgAV is believed to pathologically be related to the renal condition IgA nephropathy (IgAN), due to the same appearances in the renal histopathology together with elevated systemic IgA levels and circulating IgA immune complexes, although whether they are a spectrum of the same condition remains uncertain (9).

**Figure 1:**
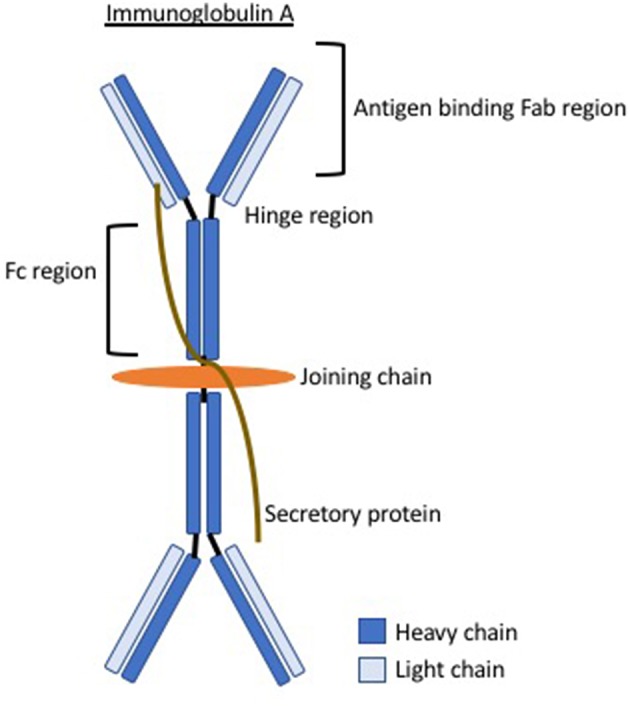
The structure of immunoglobulin A demonstrating the hinge region where abnormalities in IgA glycosylation are believed to give rise to antibody formation.

Elevated serum galactose-deficient IgA1 levels are seen in IgAV (10) and abnormal IgA1 glycosylation is believed to be a leading phenomenon in the pathophysiology. It is unknown exactly why this occurs and it is proposed that there may be abnormalities in the critical genes in the glycosylation pathway like that suggested in IgA nephropathy (11). This abnormal glycosylation results in exposure of residues, believed to arise in the hinge region of IgA (see Figure 1), this region then constitutes an antigen inducing a humoral autoimmune response. Circulating immune complexes and immune deposits contain IgA1. Galactose-deficient variants are rarely found in normal circulatory IgA1 but are much more common in patients with IgAV (or IgAN). It is unknown whether these are a temporary phenomenon during the acute phase or present even in disease quiescence. Circulating immune complexes cannot be cleared by normal mechanisms and therefore deposit inducing local tissue inflammation. For example, in the kidney, the transferrin receptor, which preferentially binds galactose-deficient IgA1, is expressed on mesangial cells and binding enhances cell proliferation, complement activation, cytokine release, and production of extracellular matrix (ECM), all of which contribute to renal inflammation. It is not certain whether the pathophysiology is entirely dependent on abnormal IgA or whether different quantities or specific abnormalities in the various galactose-deficient regions represent phenotypic differences in this condition.

### Genetic Predisposition

The geographical variation in the incidence of this condition highlights the potential role of genetic influences and IgAV is more common in the Asian population (4). There have been many genome-wide association studies that have demonstrated the influence of mutations on the predisposition to this condition and also in the way that the disease manifests itself; in particular the extent of renal involvement (due to the associated long-term renal consequences this organ has received the most attention). It is becoming apparent therefore, that genetics plays a key role in determining both the likelihood of getting the condition and also the severity of the phenotype experienced. The specific genetic risk factors for acquiring the disease that have so far been identified include genes that code for inflammatory pathways within the blood vessels themselves and those coding within the kidney (see Table 1). These include genes associated with general autoimmunity such as certain human leucocyte antigen (HLA) alleles, together with more specific ones that may influence how the vascular system responds to an insult, for example; endothelial nitric oxide synthase (eNOS), angiotensin-converting enzyme (ACE), interleukin 18 (IL18), chemokine monocyte protein chemoattractant protein (MCP), and transforming growth factor (TGF) (12). Some studies have reported protective genetic associations that reduce the risk of acquiring the disease, including certain HLA genes, which could explain ethnic differences (Table 1). With regards to specific phenotypes, most studies have focused on IgAV nephritis. The ACE, IL8, and HLA-B^*^35 genes were associated with a worse renal phenotype (12).

**Table 1:** The specific genetic risk factors that have so far been identified predisposing an individual to acquiring IgA vasculitis and those implicated in being protective against the disease [adapted from He et al. (12)].

**Genetic susceptibility**	**Genetic protection**
HLA-B*15	HLA-B*7
HLA-B*35	HLA-B*40
HLA-B*4102	HLA-B*49
HLA-B*52	HLA-B*50
HLA-A*2	HLA-A*1
HLA-A*11	HLADRB1*3
HLA-A*26	HLADRB1*7
HLA-DRB1*0103	Agtrs699M235T
HLA-DRB1*11	MEFV
HLA-DQA1*0301	PONI
HSPA21267GG	
IL1815187238-137G	
MCP1-2518TT	
MCP1-2518T	
TGF beta rs1800469-509TT	
Agt	
ACE	
C1GALT1rs	
NOS2A	
eNOS	
PONI192QQ	
MEFV	

It is therefore evident that this is not a condition associated with a single common genetic mutation, rather a combination of individual susceptibility risk factors that contribute to disease onset and severity when combined with environmental triggers.

### Environmental and Host Triggers

IgAV demonstrates seasonal tendency with fewer cases seen during the summer months supporting the theory of viral precipitants triggering the onset of this disease. This was nicely demonstrated in a very large correlation study performed in South Korea that included over 16,000 children with IgAV. The authors looked at the seasonal variation of common viruses with IgAV incidence and found temporal relationships with respiratory syncytial virus (RSV), influenza and norovirus (13). The association with viral disease may explain the tendency for the disease to be predominantly one of childhood. As IgA arises from the mucosal surfaces, several studies have looked at the role of infectious foci located within the oral cavity and the ear, nose, and throat (ENT) system. In a cohort of children in Taiwan with IgAV, 36% were found to be positive for streptococci (14). One relatively small study (*n* = 40 children with HSP) found 70% of patients had evidence of dental caries, 53% had periodontitis, rhinosinusitis in 19 (48%), tonsillitis in five (13%), and otitis media in four (10%) of the 40 patients (15). An observational drug and vaccine surveillance study (16) collected information on drug and vaccine use in children before the onset of IgAV from centers in Italy and concluded that the measles-mumps-rubella (MMR) vaccine was potentially associated with a higher risk (OR 3.4, 95% CI: 1.2–10.0) of developing IgAV. However, a much larger, European, multicenter study (17) disputed this; in 167 children with IgAV in a case-crossover study design, concluded that the OR for IgAV occurring within 3 months after vaccination was 1.6 (95% CI: 0.803.0) and hence was not significant. The analyses was stratified according by season, year of onset, infection, age, gender, and type and number of vaccines received and none of the stratifications revealed any significant associations. This suggests that vaccinations are not known to increase the risk of IgAV and hence should not be avoided.

## Clinical Presentation

IgAV usually presents in a relatively well-child and 95% of patients will present with a skin rash (18). In addition to the skin findings, the condition manifests through a classical triad of symptoms involving the gastrointestinal, musculoskeletal, and renal systems (19). Less commonly but perhaps more importantly, it can involve other systems such as the respiratory or neurological, although these are very rare.

### Skin Involvement

**Figure 2:**
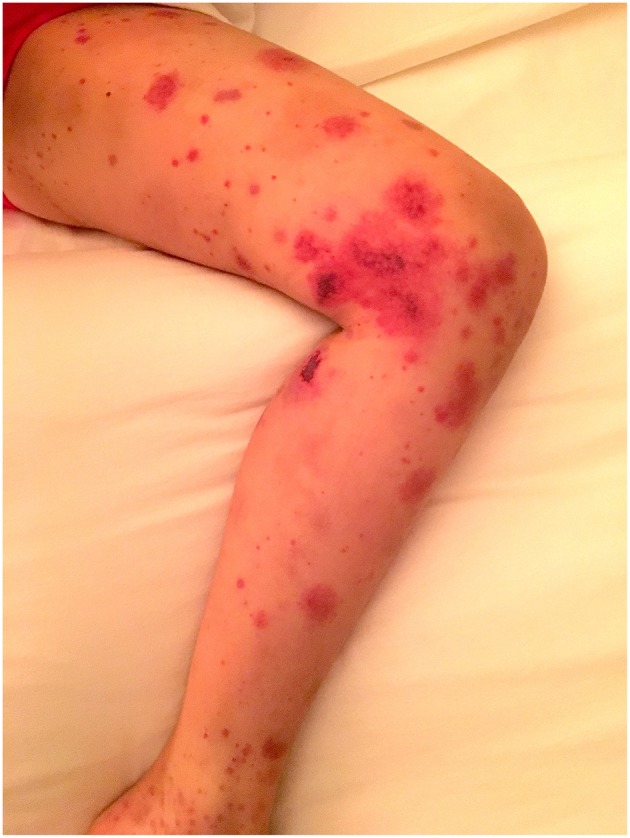
IgA vasculitis presenting in a child illustrating areas of petechiae, purpura, bruising, and necrotic lesions on the limb (parental consent obtained).

The rash is a symmetrical erythematosus petechial or purpuric rash that almost exclusively starts on the lower limbs and buttocks. It can include areas of bruising, usually intertwined with the purpura, and more rarely necrotic lesions or bullae (see Figure 2). The areas of purpura are often palpable, and the rash may extend to involve the arms and, less commonly, the trunk. Skin oedema can be located around the purpuric lesions. It is very rare to get facial involvement, although it can be seen in more severe cases but never in isolation. The diagnosis is made clinically although confirmation by histological analysis, from skin or renal biopsy, is sometimes helpful.

### Musculoskeletal Involvement

During the acute presentation, up to 70–90% of patients will have musculoskeletal involvement manifesting as either arthralgia or arthritis. The frequency of arthritis is lower at around 61–64%. Arthritis tends to have an oligo-articular pattern (4 or fewer joints), with a predilection to joints of the lower limb. Joints of the feet and ankles being most commonly involved followed by knees, wrists, elbows, and hands (14, 20, 21). The rash may be co-located in the same areas of the musculoskeletal involvement and skin oedema can mimic a swollen joint. Joint involvement can rarely precede skin involvement. Arthritis is usually transient and does not cause any residual abnormalities such as joint erosions.

### Gastrointestinal Involvement

Gastrointestinal manifestations may precede the skin manifestations, by a few days or a week, and this can sometimes lead to clinical confusion until the rash appears. Gastrointestinal (GI) tract involvement occurs in up to 72% of patients and usually presents with colicky abdominal pain, due to bowel angina (18). It can extend to include acute GI bleeding either manifesting as melaena or haematemesis that can be severe and life-threatening. Asymptomatic fecal occult blood is common and reported in 22% of patients who weren't thought to have GI involvement (14). GI bleeding has been linked to the need for a longer hospital admission (22) and in severe cases it warrants acute immunosuppressive treatment. Intussusception due to GI tract inflammation can also occur and this is a surgical emergency.

### Renal Involvement

Renal involvement, termed IgAV nephritis, or previously referred to as HSP nephritis, is usually asymptomatic and thus requires active screening. It is seen in around 40–50% of patients, most of whom have a mild renal course that self resolves (14, 18). Microscopic haematuria is the most common finding on urinalysis followed by proteinuria without oedema. Macroscopic haematuria can be seen, but it is usually short-lived in the acute phase of the disease, although some patients can evolve to have recurrent macroscopic haematuria in a similar manner to IgA nephropathy. IgAV nephritis can present as simple nephritis or nephritis associated with nephrotic syndrome (oedema, hypoalbuminaemia, and heavy proteinuria). Renal involvement is the most serious manifestation of IgAV as it is the only organ linked to long term morbidity and mortality in both childhood-onset and adult-onset disease (23). There is some evidence to suggest that patients with severe IgAV nephritis have more severe extrarenal symptoms during the acute period although much remains unknown about this condition (14).

### Other

Testicular inflammation is seen [orchiditis occurs in 14% of male patients (14)], presenting with pain and swelling that may require assessment by an experienced pediatric surgeon to exclude a testicular torsion—this distinction is important because the management of the former is conservative, and the latter is an acute surgical emergency (24). Central nervous system involvement is very rare but it is described, and manifests as seizures, weakness, confusion, visual changes, and/or reduced conscious levels (25–28).

## Diagnosis and Classification

During the 2012 International Chapel Hill Conference the eponym “Henoch-Schonlein purpura (HSP)” was decided to be replaced with IgAV based on the fact that the deposition of the abnormal IgA in the vessel wall was the most salient histopathological feature in this condition (1), although in clinical practice the term HSP is still commonly used. The 1990 American College of Rheumatology criteria were the first to attempt to develop a classification criterion for HSP. The presence of two or more of the four criteria (palpable purpura, the age of onset ≤ 20 years, acute abdominal pain and skin biopsy showing granulocytes in small arterioles or venules) provided ~87% sensitivity and specificity to distinguish from other forms of vasculitides (29). The 1990 ACR criterion was developed from a HSP cohort of 85 patients including both children and adults and other types of vasculitides like hypersensitivity vasculitis which could be misclassified as HSP as they can satisfy the two ACR criteria (purpura and biopsy findings). Hence in 2005, a new European League Against Rheumatism (EULAR)/Pediatric Rheumatology European Society (PReS) classification criterion for all childhood vasculitides including HSP was proposed based on expert consensus (30) and validated with the support of the Pediatric Rheumatology International Trials Organization (PRINTO) in 872 cases of HSP aged ≤ 18 years at disease onset. The EULAR/PReS/PRINTO criteria (31) rely on clinical features and include the mandatory presence of a vasculitic purpuric rash together with additional symptoms and signs (Table 2) yielding an excellent sensitivity (100%) and specificity (87%) in distinguishing HSP/IgAV from other types of vasculitis.

**Table 2:** The EULAR/PReS/PRINTO classification criteria for childhood IgA vasculitis.

**Criterion**	**Description**
Mandatory	Purpura or petechia with lower limb predominance
At least 1 out of 4	(1) Acute onset diffuse abdominal colicky pain (may include intussusception and gastrointestinal bleeding) (2) Histology showing leukocytoclastic vasculitis or proliferative glomerulonephritis with predominant IgA deposition. (3) Acute onset arthralgia or arthritis (4) Either proteinuria or haematuria

## Histological Diagnosis and Renal Follow up

Renal monitoring typically relies on regular urinalysis and blood pressure checks. Patients with ongoing or worsening renal inflammation should have a renal biopsy performed to determine and grade the extent of renal inflammation. Collaborative efforts across Europe have recently achieved international consensus on when to perform a renal biopsy and state that a renal biopsy should be performed if an IgA vasculitis patient has severe proteinuria (>250 mg/mmol for at least 4 weeks; although shorter duration of severe proteinuria is also a relative indication for biopsy); or persistent moderate (100–250 mg/mmol) proteinuria or impaired GFR. Due to the risk of long-term renal complications, there is consensus agreement that all patients with IgAV should have renal monitoring for at least 6 months after the acute episode, even if the initial urine is normal (32). A normal urine microscopy is indicative of an excellent long-term renal outcome even at day 7 but onset of renal disease can occur at any stage, usually within the first few months.

The International Study of Kidney Disease in Children (ISKDC) have produced histological classification criteria for IgAV nephritis and they have a role in guiding treatment. Features include the typical finding of a leukocytoclastic vasculitis of the small vessels plus any of the following; diffuse proliferation of mesangial cells and matrix without significant involvement of capillary walls or lumina and segmental necrotising lesions, endocapillary proliferation, cellular crescents, and/or inflammatory infiltrate. The ISKDC classification divides the histological appearances into six categories (I, II, III, IV, V, and VI) (33). It is most common to have early mesangial proliferative changes and if they are isolated they correspond to ISKDC histological class II, if they are combined with crescentic changes (<50%) they correspond to class III (further subdivided into a or b for focal or diffuse changes respectively), 50 -75% crescents are class IV and >75% crescents are class V. Signs of chronic damage include glomerular sclerosis, tubular loss, interstitial fibrosis, and hyaline arteriolosclerosis. On immunofluorescence IgA is seen located in the mesangium with variable degrees of accompanying IgG, IgM, and C3 staining. On electron microscopy the mesangial deposits may extend into the sub-endothelial areas and there may be subepithelial deposits. More recently modified semi-quantitative classification (SQC) scores have been proposed to enhance the sensitivity in predicting the renal outcome in IgAV, these take into account, and numerically score, certain activity, chronicity, and tubulointerstitial renal indices (33).

## Treatment

Most cases of IgAV in children spontaneously improve and do not require any specific treatment except for supportive care. In more severe cases, treatment options in part depend on the type and severity of organ involvement. Clinical trials in this area are sparse making firm evidence-based recommendations impossible although international consensus management plans have now been published (32).

### Skin Involvement

Skin involvement is usually self-limiting and doesn't require specific treatment. It may require treatment when the condition presents with a bullous or necrotic rash that jeopardizes the skin integrity, but this is rare. Proposed treatment for severe skin involvement suggested within the literature is based on single case reports or small case series, with corticosteroids recommended as the first line treatment (34–36) and should be started as soon as the bullae or concerning necrotic areas appear. They are usually given orally at a dose of ~1 mg/kg/day. Reported rare patients that have required adjunctive medications to control the lesions or minimize the exposure to corticosteroids have used Dapsone (a sulfon class of antibiotic with anti-inflammatory actions through myeloperoxidase inhibition) or azathioprine, a purine synthesis inhibitor.

### Musculoskeletal Involvement

Management of musculoskeletal involvement is usually supportive by providing pain relief usually using non-steroidal anti-inflammatory medications, such as ibuprofen. More severe cases have been reported within case series to respond to corticosteroids at a dose similar to that proposed for severe skin or GI disease (37). Additional immunosuppression is rarely required for musculoskeletal disease but any of the immunosuppressant agents including methotrexate, hydroxychloroquine and dapsone may have a role.

### Gastrointestinal Involvement

As most gastrointestinal (GI) involvement is mild and short-lived, treatment is not usually required. GI involvement that includes severe abdominal pain, GI hemorrhage, and/or intussusception will necessitate intervention. Limited evidence, suggest that abdominal pain that is not tolerable or remitting will benefit from treatment with corticosteroids as first line (usually oral at 1–2 mg/kg/day for ~2 weeks then weaned) (38). Intravenous methylprednisolone could be administered if the condition is life-threatening, oral route is not tolerated or they have failed to respond.

Second line treatments for GI disease described within the literature include the use of mycophenolate mofetil (MMF) (39), a single dose of intravenous cyclophosphamide [improved symptoms in 6 children with steroid resistant disease (40)], intravenous immunoglobulin IVIG [demonstrated efficacy in 6 out of 8 French children with GI pain, bleeding or enteropathy (41)], B cell depletion by monoclonal antibodies (7 out of 8 children with chronic steroid dependent disease achieved full remission) (42), methotrexate, colchicine and hydroxychloroquine (43). A cohort of 7 children with refractory GI bleeding that failed to respond to numerous second line drugs reportedly responded to plasma exchange and therefore this could be considered in severe, refractory cases (44).

### Renal Involvement

Treatment of renal involvement is important because it is the only organ associated with long term consequences. Clinical features (such as extent of proteinuria and renal function) combined with the renal histological appearances will facilitate treatment choices.

#### Preventing Renal Inflammation

Several large randomized controlled trials (RCTs) have been conducted in all children with IgAV focusing on the use of corticosteroids to prevent renal inflammation, including a large national RCT in the UK who compared oral corticosteroids to placebo and found no difference in terms of renal outcomes (45). The Kidney Disease: Improving Global Outcomes (KDIGO) group systematically reviewed the available literature (46, 47) and reported that compared to no therapy or supportive therapy, early treatment with corticosteroids has little, or no difference on the risk of development of kidney disease (RR 0.74, 95%CI 0.42–1.32; 5 studies, 746 participants) and little or no difference in the development of severe kidney disease (nephrotic range proteinuria, hypertension, or reduced kidney function) (RR 1.58, 95%CI 0.42–6.0). The Cochrane Systematic Review group have also reviewed the available data and concluded that there is no evidence to support the use of corticosteroids in all children with IgAV to prevent the onset of nephritis (46, 47). Updated literature reviews continue to demonstrate no trial data to support the use of drugs to prevent nephritis in all children with IgAV.

#### Established Renal Inflammation

##### Immunosuppression

There is a paucity of good quality RCT's assessing the role of immunosuppression in the treatment of histologically proven IgAV nephritis. The Cochrane collaboration concluded that there is a serious lack of evidence to support treatment for established nephritis as the research trials are few in number and generally poor quality (47). New international consensus guidelines have proposed treatment recommendations using the best available evidence and recommend oral prednisolone as first line treatment for mild renal disease (those with a normal renal function and mild/moderate proteinuria <250 mg/mmol, this usually relates to class II, or IIIa histological changes) and oral or intravenous prednisolone for moderate nephritis (<50% crescents on biopsy and impaired renal function or severe persisting proteinuria, usually histological class IIIb) together with either azathioprine, mycophenolate mofetil or intravenous cyclophosphamide. In severe nephritis (defined as >50% crescents on renal biopsy and impaired renal function or severe proteinuria >250 mg/mmol, histological class IV-V), intravenous corticosteroids and intravenous cyclophosphamide are recommended to induce remission followed by a period of maintenance treatment (48). They concluded that there doesn't appear to be a role for calcineurin inhibitors or oral cyclophosphamide in this disease.

##### Targeting the coagulation system

Abnormalities in coagulation have been implicated in the renal pathophysiology of IgAV nephritis and as such several studies have been conducted using inhibitors of the coagulation cascade. The most promising of which, was a relatively large RCT, with 89 patients, using low molecular weight heparin (administered daily over an 8-week period) alongside conventional treatment. This demonstrated improvement in the overall outcome with less ESKD and improvement in proteinuria (49). It is believed that inhibition of hyperfibrinolysis may improve the renal blood supply and hence explain the improvements that were seen. The treatment however is invasive, requiring daily subcutaneous injections and this hasn't been replicated.

##### Targeting the angiotensin system

Due to their well-recognized role in long term renal protection, it is consensus opinion that all patients should receive adjunctive ACE inhibitor (ACEi) or angiotensin receptor blocker (ARBs) for persisting proteinuria (48, 50).

##### Targeting B cells and plasma exchange

One small retrospective study has described the use of the biologic agent rituximab as a method of B cell depletion. A cohort of 8 patients with IgAV were treated with rituximab and seemingly improved with a reduction in their steroid requirement (42). It is worth noting however that large controlled trials in the similar condition IgA nephropathy have been negative, demonstrating no clinical response and that B cell depletion by monoclonal antibodies did not influence the galactose-deficient IgA or its antibodies (51). Extrapolating these findings, and assuming these conditions have similar pathophysiology, means that there is not currently an obvious role for B cell depletion in IgAV although more mechanistic studies are needed. On the contrary, plasma exchange has been used during the acute phase, as a method to remove the circulating immune complexes that occur in this disease. A cohort of 16 children with histological class III or above IgAV nephritis and a mean estimated glomerular filtration rate of 56 ml/min/m^2^ were treated with an average of 9 exchanges over 2 weeks demonstrating reported efficacy in renal parameters in 15 out of 16 patients (52). As this is an invasive procedure it is the authors' opinion that this should be reserved for the most severe, unresponsive cases.

##### Other

There is no convincing evidence to support the use of intravenous immunoglobulin (IVIG), montelukast, urokinase, vitamin E, fish oil in the treatment of IgAV nephritis. Large studies are being conducted using Chinese herbal medicines [a prospective cohort trial is underway including 600 children, (53)] although previous studies have demonstrated no long term differences and their applicability with conventional medicine is uncertain. Another potential target is the eradication of oral infections, using thorough dental hygiene and even tonsillectomy, and one study reported complete recovery in all patients following aggressive oral management (15). There are also reports of new cases of IgAV occurring as a consequence of dental treatment; as such the oral cavity is highly likely to be a trigger in some patients (54). Many groups have described the beneficial role of tonsillectomy on improving IgAV symptoms and prognosis (55–59), albeit in uncontrolled studies. The risk: benefit ratio of this invasive procedure remains uncertain although it may have a role in a select group of patients. More recent studies have begun to explore the oral microbiota and its role in IgAV pathogenesis, where differences in the microbiota composition have been seen when compared with healthy children (60). Similar changes within the gut microbiome have also been reported (61).

## Outcome

Overall the outcome is excellent in the vast majority of children (50% have spontaneous remission) however relapses can occur and there is a recognized risk of life-long renal complications. The majority of children will have a self-limiting disease course with symptoms resolving within the first 1 month and 94% of children will make a complete recovery by 2 years. Recurrent episodes of IgAV occur in around 25% of patients and there is some suggestion that they may be more common in slightly older children (aged > 8 years) and in those with nephritis (14). A study in Taiwan demonstrated that the average time interval between the first and second episodes was 9.2 months (62). It is the authors' opinion that, patients presenting with a recurrent episode should undergo renal screening again regardless of previous normal findings and they may warrant follow up to ensure complete remission occurs over time. In those with a relapsing course, immunosuppression may be a required depending on the severity of symptoms (63).

Unless patients present with severe neurological involvement, which is extremely rare, early morbidity in this condition is typically related to pain (musculoskeletal or skin) and GI symptoms. The late morbidity is almost exclusively related to renal involvement (IgAV nephritis).

The long-term consequences of IgAV nephritis include chronic kidney disease (CKD) and it accounts for 1–2% of end-stage kidney disease (ESKD) (64). Poor prognostic features indicating a worse renal outcome include a lower glomerular filtration rate (GFR) at presentation and those with nephritic or nephrotic syndrome during the acute period where the risk of progressing to some degree of chronic kidney disease (CKD) is 41% when compared to those with microscopic urine abnormalities (reported risk of CKD 15%) (65, 66).

In patients who have had a renal biopsy, around a fifth of those will continue to have significant proteinuria (>0.5 g/day) after 10 years follow up. This does not seem to correlate to the initial histological lesion or the treatment modality although there is some association to a lower level of proteinuria in patients treated with ACEi (67). In the very long term, out of the patients who have required a renal biopsy, 66% will maintain a normal renal function and normal urinalysis, and 21% will progress to ESRF within 20 years (68). A study with a median of 24 years follow up in 52 patients with childhood IgAV demonstrated additional complications with 70% of females experiencing either proteinuria or hypertension during pregnancy (69). Disease recurrence can occur in the transplanted organ in patients with IgAV who undergo a renal transplant. In a large cohort of patients from transplant centers across Belgium and France, the reported rate of disease recurrence was 12% with actuarial risk of graft loss in a first kidney graft being 2.5% at 5 years and 7.5% at 10 years (70). Despite this small but significant risk, the long-term graft outcomes are actually very good and similar to non-IgAV transplanted patients (71). Unfortunately, despite advances in medical science over the past few decades, the incidence of renal complications in IgAV hasn't decreased over time. This was demonstrated by a group in Japan who found no changes in the incidence of ESKD in IgAV during the decades from 1987–1997 to 1998–2008 (72).

## Recent Advances and Areas of Unmet Need

Recent advances highlighted in this review article include an improvement in our understanding of the pathophysiology of this condition that have resulted in its name change together with knowledge that stems from large genetic susceptibility studies, environmental population studies, and scientific exploration of IgA abnormalities. The recent publication of international consensus recommendations for the diagnosis and management of this disease will significantly standardize how we manage these children providing a platform for future trials. Practical methods that may reduce the severity of this condition include eradicating potential oral triggers, such as good dental hygiene, even considering tonsillectomy, and manipulation of the microbiota, although the data supporting these concepts is not sufficient to adjust clinical practice at present. This article has highlighted the management options available for each of the organs involved using a summary of the evidence base, consensus opinion, and author views. The use of semi-quantitative classification scores for the renal histological findings may produce improvements in the correlation between initial histological appearances and long-term renal outcome in children with IgAV nephritis.

There are many reasons that may explain why the incidence of ESKD in IgAV has not changed over time. One major limiting factor has been the lack of “standard” methods to monitor or investigate nephritis and international efforts, such as that from the SHARE consortium, should ameliorate these limitations (32). This is a challenging condition to study due to the high likelihood of a spontaneous full recovery. This condition would benefit from predefined core outcome measures for reliable comparison of studies, as very few report critical and important outcomes, such as all-cause mortality, time to ESKD, infection, malignancy, and complete remission.

Despite the majority of patients having an excellent outcome, the renal consequences remain a concern and poor outcomes haven't changed. It is our opinion that the period of renal monitoring, especially in children, provides an ideal “window of opportunity” for early intervention if we could stratify patients according to their risk of later complications. Therefore improved, early biomarkers are needed to reliably predict these patients. This condition requires urgent international collaboration using multi-center disease cohorts to allow discovery of novel biomarkers, validation of clinical recommendations, and severity scores and the development of core outcome sets. Using this set up, large scale comparisons could be made and these will provide a setting for well-conducted RCTs.

## Conclusion

IgAV is a multi-systemic common childhood vasculitis. In general, it has an excellent prognosis however there are still patients who suffer long term, typically renal, consequences from this condition. A multi-institutional, multi-speciality collaborative network is needed in order to improve the outcomes for these children with the ultimate aim to eliminate the long-term renal consequences of IgAV.

## Author Contributions

LO contributed to the construct and design of the manuscript. SS contributed to writing additional information for the manuscript.

### Conflict of Interest Statement

The authors declare that the research was conducted in the absence of any commercial or financial relationships that could be construed as a potential conflict of interest.
